# Effect of Annealing on Electrical and Optical Properties of Tin-Doped Vanadium Oxide Films for Microbolometer Applications

**DOI:** 10.3390/nano16090504

**Published:** 2026-04-22

**Authors:** Lin Cong, Mukti Rana

**Affiliations:** 1 Division of Physics, Engineering, Mathematics and Computer Sciences, Delaware State University, Dover, DE 19709, USA; 2 Research on Nanomaterial-Based Integrated Circuits and Electronics (NICE), Delaware State University, Dover, DE 19709, USA

**Keywords:** microbolometer, VO_x_, tin doped VO_x_, absorptance in the NIR region, TCR, 1/*f*-noise, infrared detector, uncooled detector, doped vanadium oxide

## Abstract

We investigate the effects of post-annealing in oxygen (O_2_) and nitrogen (N_2_) on tin-doped vanadium oxide (V_x_Sn_y_O_z_) films for microbolometer applications. The films were deposited using magnetron sputtering in an Ar:O_2_ environment. We demonstrate that low Sn doping combined with N_2_ post-annealing provides an effective approach to optimize the temperature coefficient of resistance (TCR), resistivity, and 1/*f*-noise. Compared to undoped VO_x_, V_x_Sn_y_O_z_ films exhibit an enhanced TCR, moderate resistivity, and reduced 1/*f*-noise. The 135 nm thick V_0.46_Sn_0.03_O_0.51_ film after post-annealing in N_2_ shows a TCR of −4.08%/K and a resistivity of 7.3 × 10^−2^ Ω⋅cm at 300 K, an absorptance of 63–68% in the 900–2500 nm wavelength range, and low noise voltage power spectral density (1.77 × 10^−16^ V^2^/Hz at 100 Hz under 0.3μA bias current). These results indicate that Sn-doped VO_x_ films are promising sensing materials for microbolometer applications.

## 1. Introduction

Microbolometers are uncooled infrared detectors used to detect and measure infrared radiation within specific wavelength ranges. Their resistance value changes as temperature changes. The change in resistance can be extracted as a voltage or current signal. The first bolometer was invented by Samuel P. Langley in 1878 for solar energy spectrum measurement [1]. Today, microbolometers are widely used in thermal cameras, defense and security systems, autonomous driving, medical imaging, and many other applications [2].

A general microbolometer consists of an absorber layer, a sensing layer, a support and contact arm, a 4/λ cavity where λ is the target wavelength, a mirror, and a substrate [3]. The absorber layer converts the absorbed infrared (IR) radiation to thermal energy, raising the temperature. This change in temperature leads to a change in the resistance value of the sensing layer. The cavity and the mirror form a resonant cavity that is used to improve absorption efficiency. Two of the important performance metrics of microbolometers are responsivity and detectivity.

Responsivity represents the ability of a detector to convert incident radiant power into an electrical signal. Voltage responsivity (*R_v_*) can be expressed by Equation (1).

(1) $$R_{v}=\frac{\eta \beta RI_{b}}{K_{eff}\sqrt{1+\omega^{2}\tau^{2}}}$$

where *η* is the optical absorption coefficient of the detector, *β* is the temperature coefficient of resistance (TCR) defined as the change in resistance with a change in temperature, *R* is the resistance of the detector, *I_b_* is the bias current, *K_eff_* is the effective thermal conductivity, *ω* is the modular frequency of the incident IR radiation, and *τ* is the thermal time constant [4].

Detectivity (D*) is the area-normalized signal-to-noise ratio which represents the sensitivity of the detector. It is defined as Equation (2).

(2) $$D^{*}=\frac{R_{v}\sqrt{A∆f}}{∆v_{n}}$$

where *A* is the area of the detector, Δ*f* is the noise equivalent bandwidth, and Δ*υ*_n_ is the total noise [4].

Therefore, selecting a sensing material with high TCR and absorption and low noise is critical for high-performance microbolometers. According to Voshell et al. [5], the most widely used sensing materials for microbolometers are amorphous silicon (a-Si) and its related compounds, as well as vanadium oxide (VO_x_) films. a-Si materials provide the benefits of complementary metal–oxide–semiconductor (CMOS) compatibility and a high TCR value. Rana et al. [6] reported on SiGeO films with a TCR value in the range of −3.5~−8.7%/K at room temperature. VO_x_ films exhibit suitable electrical and optical properties for the sensing layer of microbolometers. In other studies, the TCR values of VO_x_ films were reported in the range of −1.1~−7%/K at room temperature with low noise [7,8]. In addition, some new materials have been explored, such as TiO_2_ films with a TCR value of −2.8%/K [9] and carbon nanotubes with a TCR value of −2.07%/K in the low temperature range [10].

Vanadium oxide films exhibited multiple phases in different deposition techniques and conditions, which include VO(V^2+^), V_2_O_3_ (V^3+^), VO_2_ (V^4+^), and V_2_O_5_ (V^5+^) [11,12]. V_2_O_5_ is the most stable vanadium oxide phase, and it has a good TCR value, although its high resistivity results in high electrical noise. VO_2_ has a high TCR value and moderate resistivity [7]. However, its metal–isolator transition (MIT) at approximately 68 °C limits the detectable temperature range of microbolometers. Thus, the mixed phases of VO_x_ films with desirable properties are used in microbolometers [12].

In this paper, we investigate V_x_Sn_y_O_z_ films as sensing materials for thermal detectors. Here x, y, and z in V_x_Sn_y_O_z_ represent the atomic percentage of each element in the films, where x + y + z = 1. V_x_Sn_y_O_z_ films with different Sn concentrations were deposited by radio frequency (RF) magnetron sputtering technique, followed by post-annealing to obtain the Sn-doped V_2_O_5_ and Sn-doped VO_2_ films. By comparing their microstructural, electrical, and optical properties, we analyzed the influence of Sn doping in different vanadium (V) phases on the properties of V_x_Sn_y_O_z_ films.

## 2. Experimental Details

### 2.1. Film Deposition

**Figure 1:**
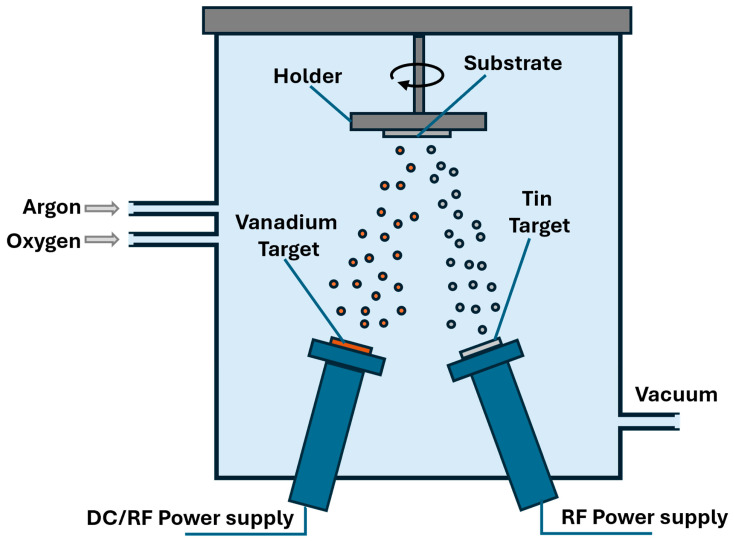
A schematic illustration of the dual-target sputtering chamber used to deposit the samples.

V_x_Sn_y_O_z_ films were deposited onto cover glasses [25 × 25 mm^2^ from VWR Scientific] and p-type Si substrates [two-inch diameter from University Wafer (South Boston, MA, USA)] by dual-target magnetron sputtering in a mixture of Ar:O_2_. The two targets are—a V target (99.9% purity, 2-inch diameter, 0.25 inches thick, from AJA International, Hingham, MA, USA) and a tin (Sn) target (99.99% purity, two-inch diameter and 0.25 inches thick from AJA international). Two sputtering systems were employed for sample deposition—a Torr sputtering system (Torr International Services LLC, NY, USA) and an AJA international sputtering system. The Torr sputtering system is equipped with two RF power supplies, and its chamber pressure is controlled by the introduced gas flow, which limits the deposition conditions. The second one is a TCR2200 UHV model sputtering system from AJA international. This system provides both direct current (DC) and radio frequency (RF) power supplies simultaneously and allows for a higher total gas flow at a stable low chamber pressure, providing the precise control of O_2_ partial pressure. Figure 1 shows a schematic illustration of the dual-target sputtering chamber used to deposit the samples. We prepared four samples, namely S0, S1, S2 and S3.

The initial depositions were done using the Torr sputtering system. The sputtering chamber was evacuated to a base pressure of ~9 × 10^−6^ Torr before sputtering. During the deposition, the chamber pressure was maintained at 4.3 × 10^−3^ Torr. The mixture gas flow consisted of 9 standard cubic centimeters per minute (sccm) of Ar and 0.3 sccm of O_2_. An RF power of 50 W was applied to the V target, while 6 W was used for the Sn target. Sample S0 was obtained after 1 h of deposition at room temperature. It is worth mentioning that 6 W is the minimum RF power required to sustain the plasma on the Sn target in the Torr sputtering system. This limitation triggered us to use the AJA International sputtering system for depositing the V_x_Sn_y_O_z_ films with different Sn concentrations. The DC sputter gun of the AJA International sputtering system was used to control the atomic concentrations of V and Sn in V_x_Sn_y_O_z_ films.

When we used the AJA international sputtering system, the base chamber pressure was ≤9.0 × 10^−6^ Torr. The deposition pressure was maintained at 3.0 × 10^−3^ Torr. The mixture gas flow consisted of 19 sccm of Ar and 1 sccm of O_2_. A DC power of 200 W was applied to the V target, and RF powers of 0 W, 10 W, and 30 W were applied to the Sn target, corresponding to samples S1, S2, and S3, respectively. The substrate temperature was kept at 450 °C. The deposition time was 40 min for all the samples.

### 2.2. Annealing

To anneal the samples, we used an AccuThermo AW610 RTP model rapid thermal annealing (RTA) system from Allwin 21 Corporation (Morgan Hill, CA, USA). We first annealed S0 in O_2_ at 450 °C for 30 min and found that after annealing, many bubbles formed. They also exhibited extremely high resistivity values (>5 Ω cm), which is not suitable for microbolometer applications. Therefore, we annealed samples S1, S2, and S3 in N_2_ at 550 °C for 40 min and compared them with S0, which was annealed in O_2_. Unless otherwise specified, samples S0, S1, S2 and S3 refer to the annealed V_x_Sn_y_O_z_ films throughout the remainder of this paper.

### 2.3. Atomic Compositions

The chemical compositions of the V_x_Sn_y_O_z_ films were determined using the energy-dispersive spectroscopy (EDS) function of a Quanta FEG 250 scanning electron microscopy (SEM) instrument from FEI (Waltham, MA, USA). An accelerating voltage of 10 kV was applied to excite the samples. The EDS detector collected the emitted characteristic X-rays to generate an energy spectrum. The chemical composition and corresponding elemental mapping were obtained through the analysis of the spectrum. The samples deposited on p-type Si substrates were used for EDS measurement to avoid interference from the O_2_ of glass substrates. The samples deposited on glass substrates were used for other tests. The samples on both silicon and glass substrates were deposited in the same deposition run.

### 2.4. Thickness and Surface Morphology 

The films’ thicknesses were measured from the step height on the deposited samples by a Bruker DektakXT stylus profilometer (Billerica, MA, USA). The surface morphology was characterized by a Bruker Innova atomic force microscope (AFM) operated in contact mode. NanoScope analysis 1.5 software was used to analyze the surface behavior of the samples.

### 2.5. X-Ray Diffraction (XRD)

The microstructural characterization of all the samples was performed using a Rigaku Ultima IV X-ray diffraction (XRD) system (Tokyo, Japan) equipped with Cu K⍺_1_ radiation (λ = 1.5406 Å) and operated at 40 kV and 44 mA. The 2θ angle scan range was set from 10° to 80°, which covers all the major phases present in vanadium oxide and tin oxide.

### 2.6. Transmission, Reflection, Absorption and Optical Bandgap

An optical measurement set up consisting of an Oriel Cornerstone 260 monochromator from Newport Corporation (Irvine, CA, USA), a light source, and an optical chopper was used to provide a specific incident light wavelength in the range of 900–2500 nm. The set up also used two Oriel 70129 model pyroelectric detectors (Newport Corporation), a SR830 model lock-in amplifier (Stanford Research System, Sunnyvale, CA, USA), and a computer to convert and record the optical signals as voltage signals at different wavelengths [13].

In the first measurement, the incident light of a particular wavelength from the monochromator directly passed through the substrate into the pyroelectric detector, and the recorded signals in the wavelength range were taken as the incident signals. After that, the incident light was passed through the films coated on substrates, where one detector collected the transmitted signals, and another detector collected the reflected signals. The transmittance (*T*) and reflectance (*R*) of the samples were calculated by dividing the transmitted and reflected signals by the incident signals, respectively. The absorptance (*A*) of the samples in the wavelength range of 900–2500 nm can be determined using Equation (3).

(3) A+T+R=1

The optical bandgap (*E_g_*) of the samples was determined using Tauc’s relation mentioned in Equation (4) [6].

(4) $${\left(\alpha hv\right)}^{\frac{1}{n}}=B(hv-E_{g})$$

where *⍺* is the absorption coefficient, *h* is the Planck constant, *υ* is frequency, *B* is a constant, and *n* is the Tauc exponent. Based on the type of dominating transition of the semiconductor, *n* is usually chosen as one of four values, *n* = 1/2 or 2/3 for direct bandgap or *n* = 2 or 3 for indirect bandgap materials [6].

The absorption coefficient (*⍺*) can be determined from the transmittance (*T*) and the reflectance (*R*) of a sample with thickness *t*. It is expressed by Equation (5) [6].

(5) $$T=\frac{{\left(1-R\right)}^{2}exp(-\alpha t)}{1-R^{2}exp(-2\alpha t)}$$

### 2.7. Resistivity and TCR Measurement

The sheet resistance (*R_s_*) of V_x_Sn_y_O_z_ films was measured at room temperature using a four-probe resistivity measuring system from Jandel (model RM3000+, Leighton Buzzard, UK). Resistivity (*⍴*) can be calculated using Equation (6).

(6) $$\rho =R_{s}\times t$$

A temperature-controlled probe station (Micromanipulator Inc. (Carson City, NV, USA)) was used to measure the resistance of the samples at different temperatures [13]. The measured temperature ranges from 10 °C to 40 °C with a recording interval of 1 °C. For sample S1 exhibiting the VO_2_ phase, resistance was also measured at 75 °C to evaluate the MIT behavior.

The TCR value represents the temperature sensitivity of films. It is expressed as a fraction change in resistance per unit change in temperature. The resistance of the films is also related to their activation energy (*E_a_*). As temperature increases, more carriers are thermally activated, leading to a decrease in resistance. This relationship can be described by the Arrhenius equation expressed by Equation (7) [6].

(7) $$R\left(T\right)=R_{0}exp(\frac{E_{a}}{kT})$$

Here, *R*(*T*) is the measured resistance value of the films at different temperatures, *R*_0_ is the pre-exponent factor that relates to the resistance value, *k* is the Boltzmann constant, and *T* is temperature [6].

It can be seen from Equation (7) that the activation energy is the slope of the Arrhenius plot of *Ln*(*R*) vs. 1/(*KT*). Thus, the TCR value of the samples can be derived as shown in Equation (8).

(8) $$TCR=-\frac{1}{R_{0}}\frac{dR}{dT}=-\frac{E_{a}}{KT^{2}}$$

### 2.8. Optical Constants

The optical constants of the V_x_Sn_y_O_z_ films were measured by an ellipsometer from J. A. Woollam (model IR-VASE Mark II, Lincoln NE, USA). The ellipsometer covers a wide spectrum ranging from 1.5 μm to 30 μm. The working principle of an ellipsometer is based on the change in polarization of light upon reflection from the film surface, which is detected and analyzed. Through the measurement of the change in the polarization of the reflected light, we can measure the amplitude ratio of p polarization and s polarization known as Ψ and the phase difference in p polarization and s polarization known as Δ. The calculation of the optical constants is very complicated, and a computer-based model provided by J. A. Woollam was used for this purpose. The model uses its algorithm to fit the measured values of Ψ and Δ to observe the wavelength dependent on the refractive index, n, and extinction coefficient, k.

### 2.9. Noise Measurement

The noise measurements of sample S0 in the as-deposited condition and after annealing in O_2_ were conducted by a 9812DX low-noise measurement system from Primarius Inc. (Shanghai, China). Noise was measured at a bias current of 0.5 μA for the frequency range of 1 Hz to 100 kHz.

Samples S2 and S3 were biased with a stable DC voltage. A 280 Ω low-noise resistor was connected in series with the samples. Since the resistance of the series was much lower than the samples’ resistance, this ensures a good voltage bias on samples. A low-noise pre-amplifier from Stanford research system SR560 (Sunnyvale, CA, USA) with a gain of 1000 was used to amplify the voltage across the series resistor. A 3562A dynamic signal analyzer from Agilent Technologies (Santa Clara, CA, USA) was used to analyze the amplified noise signal in the frequency range from 1 Hz to 100 kHz. Noise power spectral density (PSD) consisted of the 1/*f*-noise from the measured sample and the Johnson noise from the series resistor [14]. The device under test was placed in a low-frequency electromagnetically shielded box to reduce the influence from external noise. We did not determine the noise voltage PSD from S1 as the other figures of merit such as the low TCR (1.8924%/K) and formation of bubbles during the annealing process made it unsuitable for microbolometer applications.

## 3. Results

### 3.1. Atomic Composition

The energy dispersive spectroscopy (EDS) analysis indicates that the atomic percentage of each element in sample S0 changes by less than 0.3% after post-annealing in O_2_, suggesting minimal compositional variations. Samples S1, S2, and S3 were deposited and annealed under the same conditions, except for the RF power applied to the Sn target (0 W, 10 W, and 30 W), resulting in Sn concentrations of 0%, 3%, and 13%, respectively. This demonstrates that increasing the RF power on the Sn target leads to a higher Sn concentration in the films. The atomic compositions were determined to be V_0.40_Sn_0.1_O_0.5_ for S0 after annealing in O_2_, while those of S1, S2, and S3 after annealing in N_2_ were V_0.53_O_0.47_, V_0.46_Sn_0.03_O_0.51_, and V_0.37_Sn_0.13_O_0.5_, respectively.

Table 1 compares the atomic compositions of each sample with different deposition and post-deposition annealing parameters. It may be mentioned that we also compared the EDS results of S2 in the as-deposited condition and after annealing in N_2_ and found that the change in their compositions was less than 0.1% for each element. These results indicate that annealing has minimal influence on the atomic compositions of the V_x_Sn_y_O_z_ films. In addition, no nitrogen was detected in the V_x_Sn_y_O_z_ films after post-annealing in the N_2_ atmosphere, indicating that N_2_ is not incorporated into the films and N_2_ serves as an inert annealing atmosphere [15].

**Figure 2:**
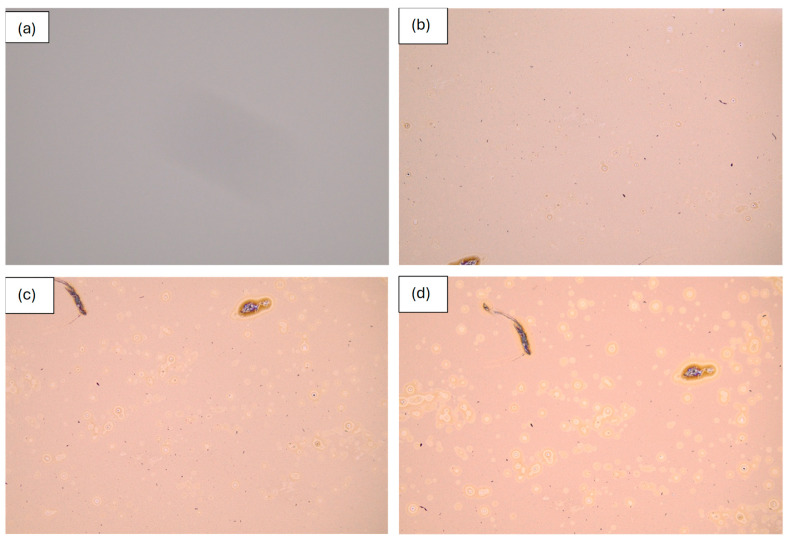
Optical microscopy images of the surface of the V_x_Sn_y_O_z_ film annealed in O_2_ at 450 °C for various durations. (**a**) As deposited, (**b**) annealed for 2 min, (**c**) annealed for 3 min, (**d**) annealed for 4 min.

Because sample S0 formed many bubbles after annealing in O_2_ for 30 min, the other V_x_Sn_y_O_z_ films deposited simultaneously with sample S0 were annealed in O_2_ for shorter durations to investigate the onset and evolution of bubble formation. Figure 2 shows the surface morphology of a V_x_Sn_y_O_z_ film annealed in O_2_ at 450 °C for various durations. Bubbles formed after 2 min of annealing in O_2_, as shown in Figure 2b. With longer annealing durations of 3 and 4 min (Figure 2c,d), both the size and density of the bubbles increased in the V_x_Sn_y_O_z_ films. Although bubbles were already present after 4 min of O_2_ annealing, the films remained amorphous. When the annealing time was extended to 30 min, the films exhibited high crystallinity, as confirmed by the subsequent XRD measurements.

**Figure 3:**
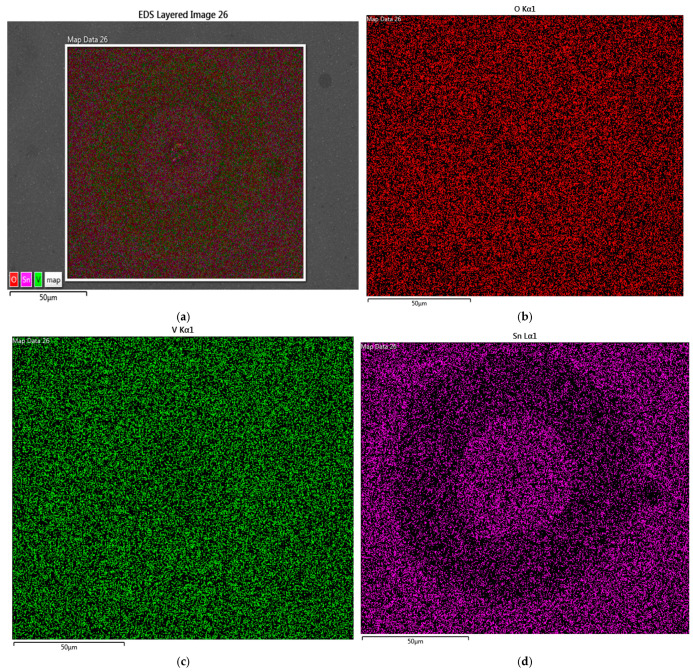
(**a**) SEM scanning area where bubble is located; (**b**) mapping of O_2_ atoms in scanning area; (**c**) mapping of V atoms in scanning area; (**d**) mapping of Sn atoms in scanning area.

Figure 3 shows the elemental mapping of a bubble in sample S0 after annealing in O_2_ for 4 min from EDS scanning. The O_2_ and V atoms are uniformly distributed within the scanning area (Figure 3b,c), whereas the Sn atoms are concentrated at the center of the bubble (Figure 3d). The results indicate that, due to the difference in Gibbs free energy between V and Sn, Sn exhibits a stronger tendency to oxidize in an O_2_ environment, which drives Sn desorption from V_x_Sn_y_O_z_ and forms the stable SnO_2_ phase [16,17]. At higher temperature, SnO_2_ acquires sufficient kinetic energy to migrate and aggregate, resulting in compositional inhomogeneity within the film. The mass transport, phase separation, and stress evolution may contribute to the formation of bubbles [18,19]. Under a nitrogen atmosphere, the limited external oxygen supply suppresses extensive oxidation. Based on the observed bubbles and the Sn aggregation at their centers, the formation of bubbles disrupts the uniformity of Sn within the films.

### 3.2. Thickness and Surface Morphology

**Figure 4:**
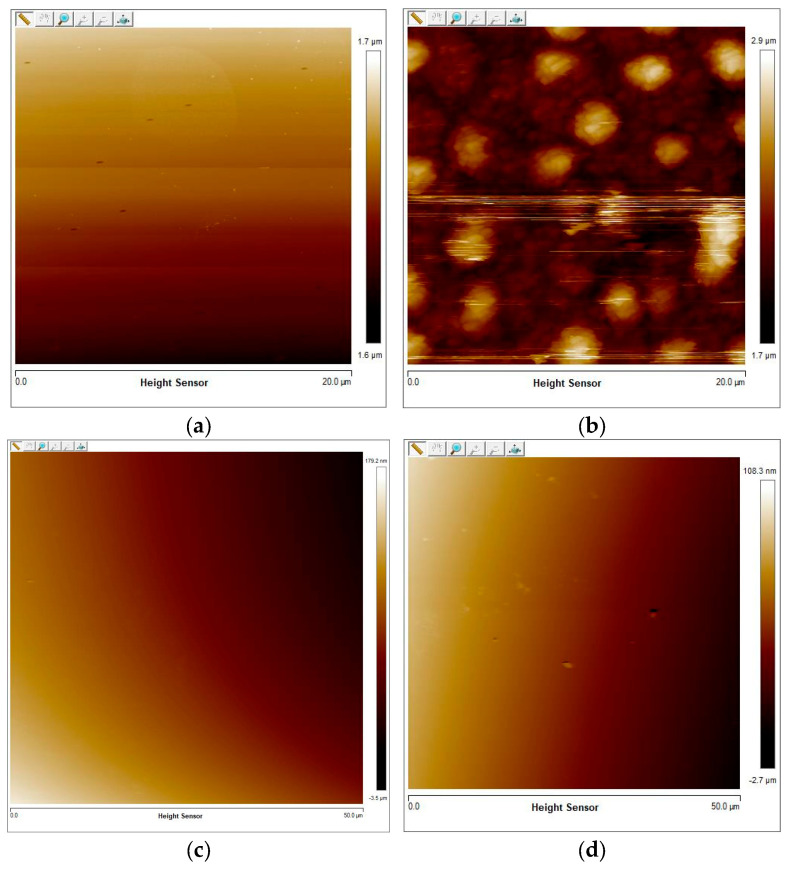
Surface morphology of samples: (**a**) S0 as deposited; (**b**) S0 annealed at 450 °C for 40 min in O_2_; (**c**) S2 as deposited at 450 °C; (**d**) S2 annealed at 550 °C for 40 min in N_2_.

The thickness of sample S0 under the as-deposited condition was 151.2 nm, and it was increased to 645 nm after annealing at 450 °C for 40 min in O_2_. Figure 4a,b exhibit the surface morphology of S0 under the as-deposited and after annealing conditions, respectively. The average surface roughness (*R_a_*) of the as-deposited film was 2.63 nm, and the root mean square roughness (*R_s_*) was found to be 3.5 nm. After annealing, the *R_a_* value was increased to 139 nm, and the *R_s_* value was increased to 180 nm. Figure 4b also shows the island-like structure on the surface of sample S0 after annealing in O_2_.

The thickness of sample S2 was 120 nm under as-deposited conditions at 450 °C, and it increased to 135 nm after annealing at 550 °C for 40 min in N_2_. Figure 4c,d compare the surface morphology of sample S2 before and after annealing. The AFM scanning area corresponding to this was 50 μm × 50 μm. Before annealing, sample S2 had an *R_a_* of 6.9 nm and an *R_S_* of 8.4 nm. After annealing, *R_a_* increased to 7.07 nm, and *R_S_* increased to 17.3 nm. Table 2 shows the thickness and surface roughness of samples before and after annealing.

The film thickness and roughness results indicate that post-annealing increases both the thickness and surface roughness of the V_x_Sn_y_O_z_ films. The influence of annealing in N_2_ on the surface morphology was much less prominent than annealing in O_2_. Post-annealing changes the lattice strain and crystallinity of the V_x_Sn_y_O_z_ films, which increases their thickness and surface roughness [15,20,21]. Regarding the island-like structure observed on sample S0 after annealing in O_2_, the analysis combined with the EDS mapping results from Figure 3 suggests that these features were likely to be caused by Sn aggregation during the post-annealing process. For V_x_Sn_y_O_z_ films annealed in O_2_, Sn aggregation disrupted the uniformity of Sn doping in the V_x_Sn_y_O_z_ films. This nonuniform Sn doping in V_x_Sn_y_O_z_ films made them unsuitable for the sensing layer of microbolometers. We did not perform any analysis on the regions where we found bubbles.

### 3.3. XRD Measurement

**Figure 5:**
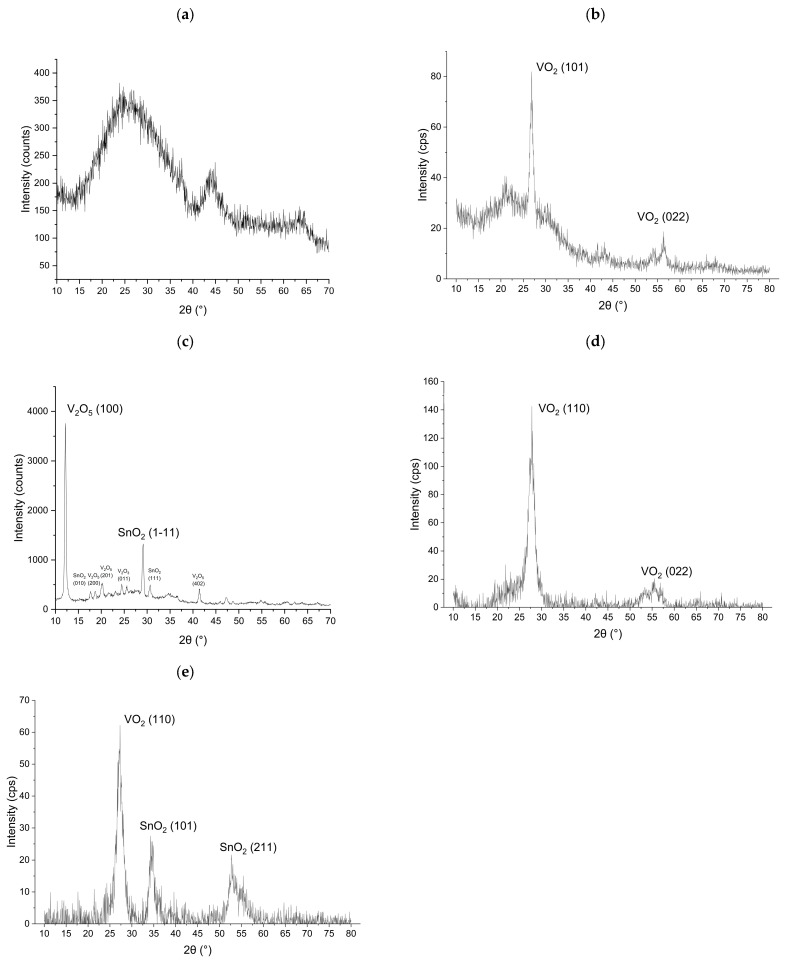
XRD pattern of samples: (**a**) S0 as deposited; (**b**) S0 after annealing in O_2_; (**c**) S1 after annealing in N_2_; (**d**) S2 after annealing in N_2_; (**e**) S3 after annealing in N_2_.

All the as-deposited samples exhibit an amorphous microstructure. An example is provided by sample S0 as deposited, as shown in Figure 5a. During the annealing process, thermal energy activates atomic diffusion within the films, and the V and Sn atoms combine with O_2_ and form crystalline phases [15]. As shown in Figure 5b, after annealing in O_2_, distinct peaks appear in the XRD pattern of sample S0, particularly at 2θ = 12.185° and 29.071°. X-ray phase analysis indicates that these peaks correspond to the V_2_O_5_ phase with a [100] orientation at 12.185° and the SnO_2_ phase with a [111] orientation at 29.071°. The presence of multiple peaks confirms that sample S0 became a polycrystalline film after annealing.

Figure 5c,d show that samples S1 and S2, after annealing in N_2_, contain VO_2_ phases. XRD analysis confirms that the crystal structure of VO_2_ is monoclinic [22]. The main VO_2_ peak appears at 26.83° for S1 and shifts to 27.85° for S2, both corresponding to the [110] orientation. With Sn doping, the VO_2_ peak shifts to higher angles and shows an increased full width at half maximum (FWHM) value, indicating enhanced lattice distortion and strain, which lead to a reduction in interplanar spacing (d-value) and crystallite size [23].

As Sn concentration increases in V_x_Sn_y_O_z_ films, sample S3 exhibits secondary SnO_2_ phases, with peaks appearing at 2θ = 34.59° and 52.82°, corresponding to the rutile tetragonal structure [24], as shown in Figure 5e. The formation of the SnO_2_ phases indicates partial Sn segregation from the VO_2_ lattice. The VO_2_ peak of S3 is observed at 27°. The increased crystallite size of VO_2_ demonstrates that Sn incorporation initially reduces the d-value and crystallite size, while phase separation in the V_x_Sn_y_O_z_ films with high Sn concentration alters this trend. The SnO_2_ phase disrupts the lattice continuity and uniformity of the V_x_Sn_y_O_z_ films, leading to the formation of phase boundaries.

### 3.4. Absorption and Bandgap

**Figure 6:**
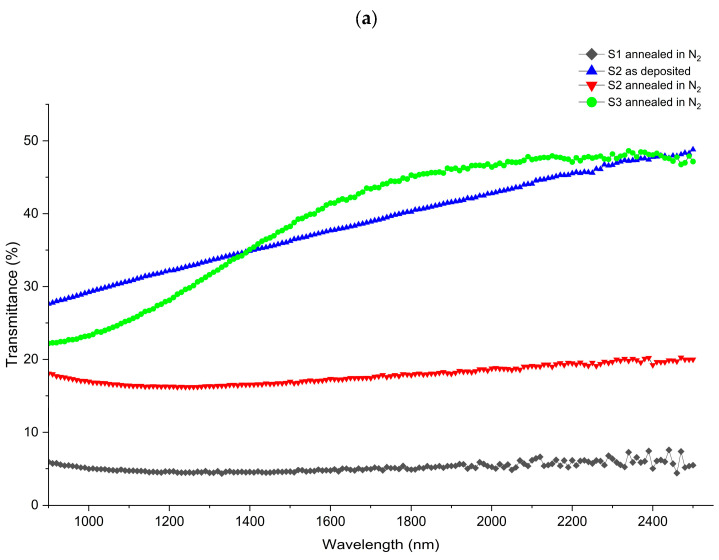

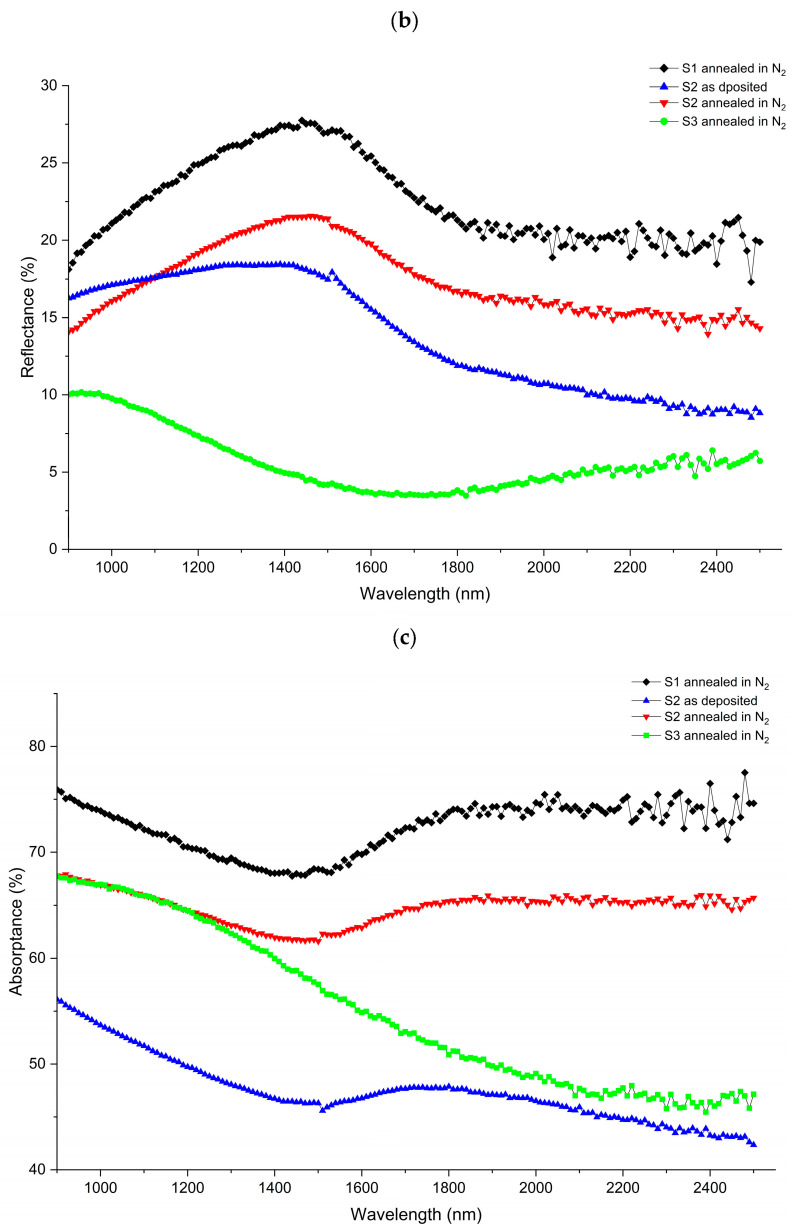
Optical properties of various samples: (**a**) transmittance, (**b**) reflectance, (**c**) absorptance.

The formation of bubbles in sample S0 disrupts the uniformity of Sn doping in V_x_Sn_y_O_z_ films. Therefore, the optical properties (transmittance, reflectance, and absorptance) of samples S1, S2, and S3 are compared in the near-infrared (NIR) region (900–2500 nm) at room temperature excluding S0. In addition, the data for sample S2 before annealing were collected under the same conditions to evaluate the effect of post-annealing in N_2_ on the V_x_Sn_y_O_z_ films, as shown in Figure 6.

Figure 6a shows that the transmittance of the V_x_Sn_y_O_z_ films in the NIR region increases with increasing Sn concentration. Sample S3 (V_0.37_Sn_0.13_O_0.5_), which contains the highest Sn concentration among the three samples, exhibits the highest transmittance, ranging from 22.5% to 47%. In contrast, sample S1 (V_0.53_O_0.47_) shows a much lower transmittance of approximately 5%. For sample S2 (V_0.46_Sn_0.03_O_0.51_) under as-deposited conditions, the transmittance is 27.64% at a wavelength of 900 nm and increases to 48.82% at 2500 nm. After annealing in N_2_, the transmittance of sample S2 decreases to approximately 17.5% over the same wavelength range. These results indicate that annealing in N_2_ decreases the transmittance of V_x_Sn_y_O_z_ films in the NIR region.

Figure 6b shows that the reflectance of V_x_Sn_y_O_z_ films in the NIR region decreases as Sn concentration increases in the films. Sample S3 exhibits the lowest reflectance, remaining below 10%, whereas sample S1 displays the highest reflectance, ranging from 17.5% to 27.5%. For sample S2, reflectance before annealing ranges from 8.7% to 18.4%, while after annealing it increases to 16.3–20.2%. Annealing in N_2_ increases the reflectance of V_x_Sn_y_O_z_ films in the NIR region.

Figure 6c presents the absorptance of V_x_Sn_y_O_z_ films with different Sn concentrations. After annealing, sample S2 with low Sn doping (3%) exhibits an absorptance of 63–68% in the NIR region, representing an increase of approximately 8–26% compared with S2 before annealing (42.3–56.1%). However, this absorptance remains lower than that of sample S1, which exhibits values of 68% to 75% over the same wavelength range. Both samples S1 and S2 after annealing in N_2_ exhibit a similar trend: absorptance decreases in the short wavelength region (900~2000 nm) and increases in the long wavelength region (2000~2500 nm). Sample S3 shows absorptance values similar to sample S2 in the wavelength range of 900–1500 nm, but it exhibits a decreasing trend in the long wavelength range (1500 nm to 2500 nm).

**Figure 7:**
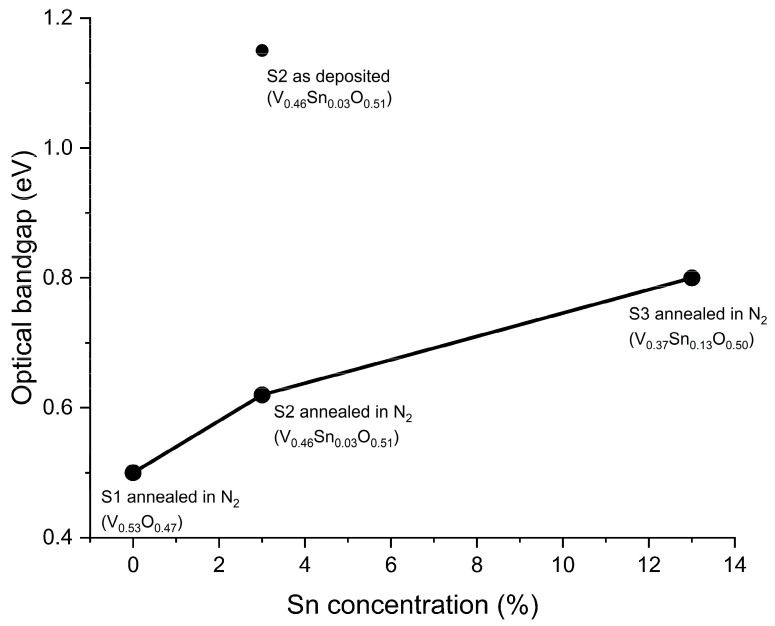
Optical bandgap of V_x_Sn_y_O_z_ films with different Sn concentrations.

All samples are found to have a direct optical bandgap. Figure 7 shows the optical bandgap of V_x_Sn_y_O_z_ films with different Sn concentrations. The optical bandgap of sample S1 (V_0.53_O_0.47_) is 0.5 eV. Yin et al. [25] reported that the optical bandgap of VO_2_ is ~0.5 eV at room temperature, which is consistent with the value obtained for sample S1. For sample S2 (V_0.46_Sn_0.03_O_0.51_), the optical bandgap is 1.15 eV for the as-deposited condition, and it decreases to 0.62 eV after annealing in N_2_. Sample S3 (V_0.37_Sn_0.13_O_0.5_) exhibits an optical bandgap of 0.8 eV.

N_2_ annealing promotes the transformation of amorphous V_x_Sn_y_O_z_ films into a crystalline VO_2_ phase. With improved crystallinity, the structure becomes more ordered, which leads to a decrease in the optical bandgap and changes in optical properties [26]. Photon energy (*E*) is inversely proportional to wavelength (λ), as expressed by Equation (9).

(9) $$E=\frac{hc}{\lambda }$$

where, *h* is Planck’s constant, and *c* is the speed of light.

When the incident photon energy exceeds the bandgap, band-to-band transitions become more probable, corresponding to intrinsic absorption [27]. Therefore, a decreased optical bandgap increases the wavelength range of intrinsic absorption, resulting in an increase in absorptance.

Sample S1 has a low optical bandgap of 0.5 eV, and its intrinsic absorption range covers the measured NIR region. EDS analysis indicates that S1 is a non-stoichiometric VO_x_ film with an oxygen concentration of 47%, consistent with a high concentration of oxygen vacancies or defects. The oxygen vacancies introduce additional energy levels within the bandgap, leading to impurity and defect-related absorptions [28]. Dual optical absorption consists of intrinsic absorption and absorption from impurities and defects, resulting in high absorptance in the NIR region [29,30]. With low Sn doping (sample S2), EDS result shows an increase in oxygen concentration from 47% in S1 to 51% in S2, leading to an increase in the optical bandgap and a decrease in dual absorption in the NIR region. The increased optical bandgap results in increased transmittance and decreased reflectance in the NIR region.

Under high Sn doping (sample S3), XRD analysis shows the formation of SnO_2_ phases. SnO_2_ is a wide-bandgap material (~3.6 eV), which increases the overall bandgap of the film and introduces structural inhomogeneity in the film [31]. Compared to samples S1 and S2, the high transparency of the SnO_2_ phases enhances transmittance and reduces reflectance. Sample S3 exhibits an optical bandgap of 0.8 eV, and its absorptance is similar to S2’s in the wavelength range of 900–1550 nm, indicating that dual absorptance dominated in this region. At longer wavelengths, where the photon energy is lower than the optical bandgap, absorptance decreases with increasing wavelength.

Overall, with increasing Sn concentration, the optical bandgap of V_x_Sn_y_O_z_ films increases. In the NIR region, transmittance increased, reflectance decreased, and absorption decreased.

### 3.5. Resistivity and TCR

Table 3 summarizes the room temperature resistivity of the V_x_Sn_y_O_z_ films. Sample S0 exhibits the highest resistivity of 5.24 Ω⋅cm, because its dominant phase is V_2_O_5_, which has the largest resistivity among vanadium oxides [32,33]. The resistivity of samples S1, S2, and S3 increases with Sn concentration in the V_x_Sn_y_O_z_ films, with measured values of 6.19 × 10^−2^ Ω⋅cm, 7.3 × 10^−2^ Ω⋅cm, and 2.47 × 10^−1^ Ω⋅cm, respectively.

**Figure 8:**
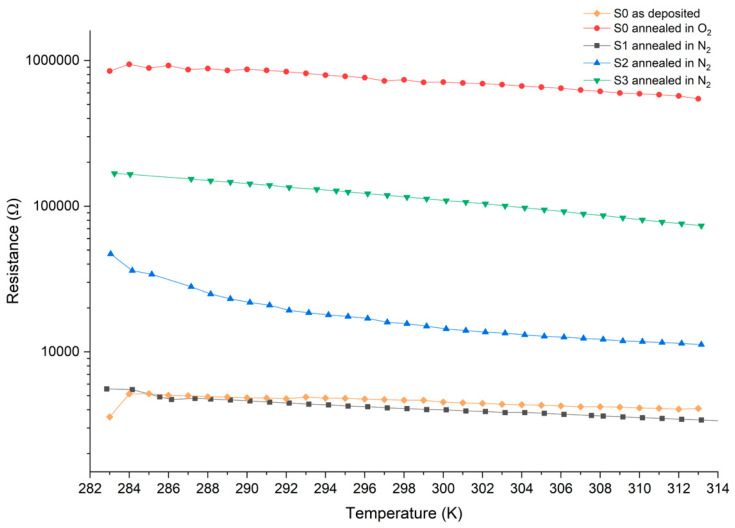
The resistance value of samples at a temperature from 283 K to 313 K.

Before annealing, the amorphous V_x_Sn_y_O_z_ films exhibit low resistivity. As shown in Figure 8, sample S0 before annealing has a low resistance (≤5.1 kΩ) in the temperature range of 10–40 °C (283–313 K), which is comparable to the resistance of sample S1 annealed in N_2_, a VO_2_ phase-based VO_x_ film. After annealing in either N_2_ or O_2_, the resistance of V_x_Sn_y_O_z_ films increases, as shown in Figure 8. In particular, sample S0 annealed in O_2_ forms a V_2_O_5_ phase, resulting in a significantly higher resistance of 546–943 kΩ over the same temperature range.

When comparing the VO_2_ phase-based samples—S1, S2, and S3—the variation in resistivity can be explained by considering the combined effects of Sn doping on O_2_ vacancy concentration and carrier transport. The resistivity of semiconductor materials depends on free carrier concentration and carrier mobility. In VO_2_, vanadium exists in the V^4+^ oxidation state, corresponding to a 3d^1^ electronic configuration. The EDS results show that the O_2_ atomic concentration in all samples is 50 ± 3%, indicating the presence of a certain amount of O_2_ vacancies, which lead to the formation of V^2+^ (3d^3^) or V^3+^ (3d^2^). It is well known that an increase in O_2_ vacancies in films gives rise to free electron density and decreases resistivity and the TCR [30]. Guantt et al. [34] reported that VO_x_ (0.8 < x < 1.3) films with a face-centered cubic (FCC) microstructure have a resistivity ranging from 1 × 10^−3^ to 6.8 × 10^4^ Ω⋅cm and a TCR varying from 0 to −4%/K.

The introduction of Sn^4+^ (4d^10^) into the films lead to the partial substitutions of V^2+^/V^3+^ or even V^4+^, resulting in a reduction in O_2_ vacancies and decrease in free electron concentration [35,36]. In addition, Sn doping in the films results in changes in lattice strain and the VO_2_ crystal structure due to the difference in the ionic radius between Sn^4+^ and V^4+^ [37]. These structural modifications restrict the mobility of free carriers, particularly when the SnO_2_ crystalline phase forms. The difference in the bandgap of VO_2_ (~0.5 eV) [25] and SnO_2_ (~3.6 eV) [31] results in structural inhomogeneity in the V_x_Sn_y_O_z_ films, which reduces carrier mobility [38,39]. As a result, the resistivity of the VO_2_-based V_x_Sn_y_O_z_ films increases with increasing Sn doping concentration.

Because VO_2_ has an MIT property at around 68°C [7], where the VO_2_ phase changes from a semiconductor to a metallic phase, we measured the resistance value of S1 at 75 °C (348 K), and no MIT behavior was observed. Liu et al. [37] reported that the MIT in VO_2_ follows the Peierls–Mott mechanism. The crystal structure of VO_2_ is the rutile (R) type at high temperature, in which V-V chains form a one-dimensional arrangement with equal spacing along the c direction, resulting in the metallic phase [40]. The rutile (R) structure transforms into the monoclinic M1 phase, where lattice distortion leads to V-V pairing (dimerization), opening a bandgap. V^4+^ has a 3d^1^ electronic configuration, and the strong Coulomb repulsion between electrons results in the bandgap increasing, which is the Mott mechanism.

The MIT temperature of VO_2_ decreases with increasing oxygen vacancy concentration and can eventually disappear, with the system exhibiting metallic-like behavior, because oxygen vacancies act as electron donors [41]. This is consistent with the measured resistivity of samples. The XRD results indicate that low Sn doping induces lattice distortion due to the difference in the ionic radius between V^4+^ and Sn^4+^. For the films with high Sn concentration, Sn doping leads to the formation of a secondary SnO_2_ phase with a rutile tetragonal structure, resulting in polycrystalline and multiphase films. The combined effects of oxygen vacancies, Sn substitution, and secondary phase formation disrupt the Peierls–Mott mechanism, leading to the suppression or disappearance of the MIT behavior [30,37].

**Figure 9:**
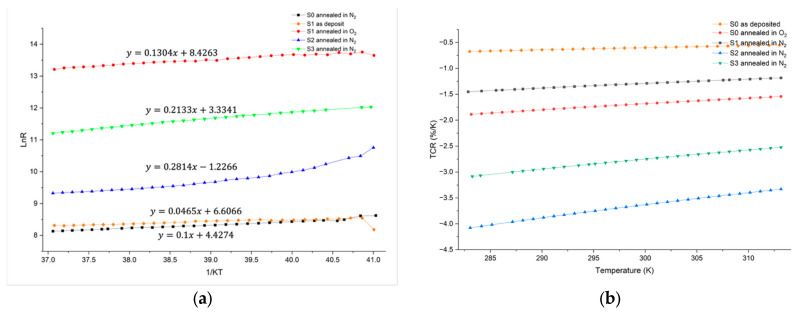
(**a**) ln(R) vs. 1/(KT) plots and (**b**) variations in TCR for samples in temperature range from 283 K to 313 K.

The activation energy of each sample was determined by the relationship between *Ln*(*R*) and 1/(*KT*), and the TCR was calculated using Equation (8). As shown in Figure 9, sample S0 (V_0.4_Sn_0.1_O_0.5_) after annealing in O_2_ forms a V_2_O_5_ phase-based V_x_Sn_y_O_z_ film, exhibiting an activation energy of 0.1304 eV and a TCR value of −1.89%/K at room temperature. These values are significantly higher than those of S0 before annealing, which shows an activation energy of 0.0465 eV and a TCR of −0.67%/K. However, these values remain lower than those of the V_x_Sn_y_O_z_ films (S2 and S3) annealed in N_2_ that are dominated by the VO_2_ phase. This comparison indicates that post-annealing effectively enhances both the activation energy and TCR of V_x_Sn_y_O_z_ films, and the VO_2_ phase-based V_x_Sn_y_O_z_ films exhibit higher activation energy and TCR.

Among samples S1, S2, and S3, which are VO_2_ phase-based V_x_Sn_y_O_z_ films, undoped sample S1 exhibits a relatively low activation energy of 0.1 eV and TCR value of −1.45%/K at room temperature. Its TCR is lower than those reported for other VO_x_ films (−1.1~−7%/K) [7,8]. This behavior is likely attributed to relatively low crystallinity of sample S1 and its lower O_2_ concentration (47%) [34].

High-Sn-doped sample S3 (V_0.37_Sn_0.13_O_0.5_) has an activation energy of 0.2133 eV and a TCR value of −3.08%/K at 300 K. In contrast, low-Sn-doped sample S2 (V_0.46_Sn_0.03_O_0.51_) shows the highest activation energy of 0.2814 eV and the highest TCR value of −4.08%/K at 300 K among all samples. These results indicate that for VO_2_ phase-based V_x_Sn_y_O_z_ films, activation energy and the TCR decrease with increasing Sn concentration in films.

Most of the reported TCR values of VO_x_ or V_2_O_5_/V films for microbolometer applications range from −0.4%/K to −4%/K [8,12,15,34,42]. Rana et al. [6] reported on amorphous SiGeO films with TCR values ranging from −3.5%/K to −8.7%/K. Kwon et al. [9] reported on TiO_2_ films for microbolometer applications with a TCR value of −2.8%/K and resistivity ranging from 0.01 to 10 Ω⋅cm. Compared with these reported sensing materials for microbolometer applications, low-Sn-doped sample S2 (V_0.46_Sn_0.03_O_0.51_) exhibits a relatively high TCR value of −4.08%/K with a resistivity of 0.073 Ω⋅cm, demonstrating its potential for microbolometer sensing applications.

### 3.6. Optical Constant

Sample S1 is an undoped VO_x_ film, which was deposited primarily as a reference to compare the TCR and resistivity of V_x_Sn_y_O_z_ films prepared under the same deposition conditions as samples S2 and S3. Considering the low TCR obtained from sample S1, which made this film not suitable for microbolometer applications, we did not perform experiments to determine the optical constants and noise measurement for this sample.

**Figure 10:**
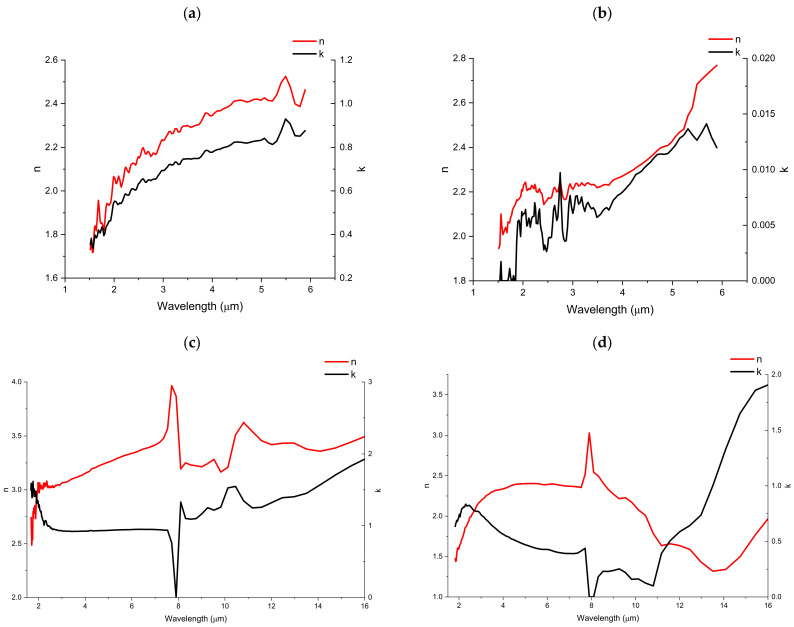
Optical constants of samples (**a**) S0 as deposited, (**b**) S0 after annealing in O_2_, (**c**) S2 after annealing in N_2_, and (**d**) S3 after annealing in N_2_.

Figure 10a shows that the refractive index (*n*) of sample S0 (as deposited) varied from 1.72 to 2.53, and the extinction coefficient (*k*) varied between 0.34 and 0.93 in the wavelength range from 1.5 μm to 6 μm. After annealing in O_2_, the refractive index increased to 1.92–2.86, while the extinction coefficient decreased to 0–0.014 (Figure 10b). However, since many random bubbles formed in sample S0 after annealing in O_2_, the measured optical constants may be affected by increased measurement uncertainty.

As shown in Figure 10c, sample S2 shows a refractive index (*n*) ranging from 2.5 to 3.5 and an extinction coefficient (*k*) ranging from 0 to 1.9 in the wavelength region of 1.6–16 μm. Sample S3, shown in Figure 10d, displays a refractive index (*n*) of 1.44–3.0 and an extinction coefficient (*k*) of 0–1.92 in the same wavelength region. Compared with S2, the high-Sn-doped V_x_Sn_y_O_z_ film (S3) exhibits a lower refractive index (*n*) and extinction coefficient (*k*). Both samples show a jump in the refractive index (*n*), while the extinction coefficient (*k*) approaches zero near the wavelength of 8 μm. This indicates that the films exhibit very high reflectance and transmittance, with low or 0 absorption when the incident light wavelength is around 8 μm, because of the large refractive index (*n*) and 0 extinction coefficient (*k*).

Sample S2 exhibits a nearly constant extinction coefficient (*k*) of ~0.9 in the wavelength range of 2.7–7.7 μm, and sample S3 also shows a slight variation in this range. This indicates that the V_x_Sn_y_O_z_ films possess stable absorptance in this wavelength range. The VO_x_ films were reported with a refractive index (*n*) of 1.85–2.7 [43,44,45] and an extinction coefficient (*k*) of 0.01 to 0.2 [43,46]. In comparison, Sn doping in the V_x_Sn_y_O_z_ films increases both the refractive index (*n*) and the extinction coefficient (*k*).

### 3.7. Noise

Johnson noise originates from the random thermal motion of the charge carriers in a resistor, and the noise voltage PSD of Johnson noise is expressed by Equation (10) [4].

(10) $$s_{v}(f)=4k_{B}TR$$

where, *k_B_* is the Boltzmann constant, and *R* is the resistance of the film at temperature *T* (Kelvin).

Moreover, 1/*f*-noise is the dominant noise source at low frequencies and typically surpasses other noise types. There is still debate regarding the origin of 1/*f*-noise, and it is commonly observed in all electrical devices. Hooge indicated that 1/*f*-noise is a bulk effect, and the major noise source exists at low frequency (<1 kHz) [47].

**Figure 11:**
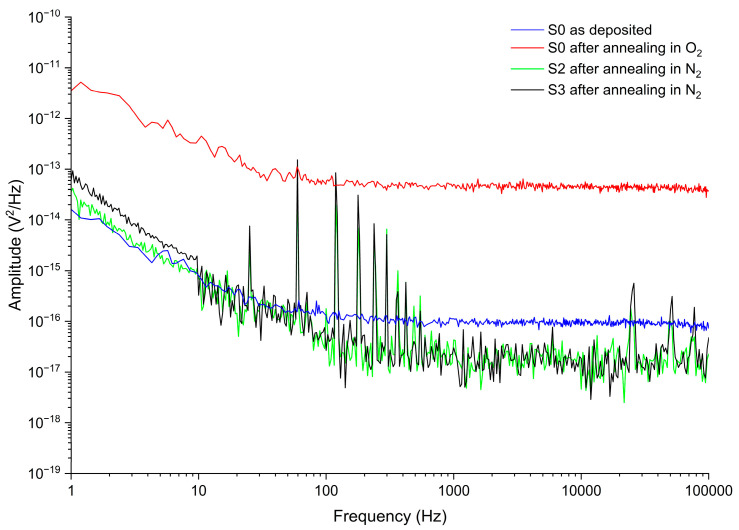
Noise voltage PSD of various samples.

Figure 11 shows the noise voltage PSD of samples S0, S2, and S3 in the frequency range of 1 Hz–100 kHz under a bias current of 0.5 μA. Johnson noise corresponds to the flat portion of the spectrum and is determined after 1 kHz. The value of the Johnson noise voltage PSD is in accordance with the 280 Ω resistor, which can be calculated by Equation (10).

Sample S0 under the as-deposited condition exhibits a noise voltage PSD decreasing from 1.81 × 10^−14^ V^2^/Hz at 1 Hz to 1.32 × 10^−16^ V^2^/Hz at 100 Hz. After annealing in O_2_, the noise voltage PSD increases by approximately two orders of magnitude, which is attributed to the formation of the V_2_O_5_ crystalline phase with a much higher resistivity [47].

Sample S2 (V_0.46_Sn_0.03_O_0.51_) exhibits a lower 1/*f*-noise. For this sample, the noise voltage PSD decreases from 3.66 × 10^−14^ V^2^/Hz at 1 Hz to 1.77 × 10^−16^ V^2^/Hz at 100 Hz. The noise voltage PSD of sample S3 (V_0.37_Sn_0.13_O_0.5_) is measured as 6.03 × 10^−14^ V^2^/Hz at 1 Hz to 2.66 × 10^−17^ V^2^/Hz at 100 Hz.

Abdel-Rahman et al. [48] reported a voltage noise PSD of ~1.32 × 10^−9^ V^2^/Hz at 12 Hz with a bias current of 11.41 μA for the vanadium oxide films formed by the alternate V_2_O_5_ and V layers. Kumar et al. [49] reported that the total noise per unit bandwidth of vanadium oxide (mixture of V_2_O_5_ and VO_2_ phases) films at 50 μA bias current was 2.3 μV//√Hz. By comparing these results with our measurements, it is evident that Sn doping significantly reduces the 1/*f*-noise of the VO_x_ films. Moreover, maintaining a low Sn concentration and avoiding the formation of the SnO_2_ phase in the V_x_Sn_y_O_z_ films can decrease 1/*f*-noise, as evident from the graph.

Based on the Hooge model, *1*/*f*-noise was determined by the quality of crystal and the scattering mechanisms that determine the mobility [47]. Under low Sn doping, the reduction in O_2_ vacancies and related defects leads to a lower 1/*f*-noise in V_x_Sn_y_O_z_ films. At the same time, compared with the VO_x_ film reported by Abdel-Rahman et al. [8], which exhibits a resistivity of 0.047 Ω⋅cm and a TCR of −3.18%/K, sample S2 shows a slightly higher resistivity of 0.073 Ω⋅cm. This indicates that low Sn doping does not induce significant changes in carrier density and mobility in VO_x_ films.

With increasing Sn concentration in V_x_Sn_y_O_z_ films, particularly when a secondary SnO_2_ phase forms, such as sample S3, resistivity increases to 0.247 Ω⋅cm, resulting in a moderate increase in 1/*f*-noise compared with low-Sn-doped films (S2). Table 4 compares the electrical and optical properties of sample S2 (V_0.46_Sn_0.03_O_0.51_) with other reported sensing materials used for microbolometer applications.

## 4. Conclusions

Post-annealing in N_2_ can effectively modify the optical and electrical properties of V_x_Sn_y_O_z_ films. Compared with the as-deposited amorphous V_x_Sn_y_O_z_ films, the VO_2_ phase-based V_x_Sn_y_O_z_ films obtained after post-annealing in N_2_ exhibit a reduced optical bandgap, decreasing from 1.15 eV to 0.62 eV. The change in the optical bandgap increases the absorptance of V_x_Sn_y_O_z_ films in the NIR region, with a maximum increase of up to 26%. Meanwhile, post-annealing leads to a slight increase in resistivity and a significant increase in the TCR while having minimal impact on 1/*f*-noise. In contrast, the V_x_Sn_y_O_z_ films after post-annealing in O_2_ show increased total noise due to the formation of the high resistivity of V_2_O_5_. In addition, bubble formation during post-annealing in O_2_ severely disrupts film uniformity. As a result, the annealing of V_x_Sn_y_O_z_ films in O_2_ is not suitable for microbolometer applications.

Compared with reported VO_x_ (1.3 ≤ x ≤ 2) films, the low-level Sn-doped V_x_Sn_y_O_z_ film (sample S2) exhibits a relatively high TCR value of −4.08%/K and a low resistivity of 0.073 Ω⋅cm while suppressing the MIT at 75 °C. However, as the Sn concentration increases in V_x_Sn_y_O_z_ films, such as in sample S3, the formation of a secondary SnO_2_ phase leads to an increase in resistivity (0.247 Ω⋅cm) and a decrease in the TCR value (−3.08%/K). Sn doping effectively reduces 1/*f*-noise in VO_x_ films. Although increasing Sn concentration leads to higher resistivity and a slight increase in total noise, both low- and high-Sn-doped V_x_Sn_y_O_z_ films still exhibit lower 1/*f*-noise compared to undoped VO_x_ films. V_0.46_Sn_0.03_O_0.51_ exhibits lower 1/*f*-noise. For this sample, the noise voltage PSD decreases from 3.66 × 10^−14^ V^2^/Hz at 1 Hz to 1.77 × 10^−16^ V^2^/Hz at 100 Hz. Sn doping in VO_2_ phase-based VO_x_ films also increases transmittance in the NIR region while reducing absorptance. However, at low Sn concentration (V_0.46_Sn_0.03_O_0.51_), the V_x_Sn_y_O_z_ films still maintain high absorptance (≥65%) in the NIR region.

Overall, low Sn doping combined with post-annealing in N_2_ provides an effective approach to optimizing the optical and electrical properties of V_x_Sn_y_O_z_ films for microbolometer applications. The films exhibit a high TCR, strong NIR absorptance, and low 1/*f*-noise, enabling the improved responsivity and detectivity of thermal detectors. In future work, we will focus on evaluating microbolometer performance using V_x_Sn_y_O_z_ films as sensing materials.

## Figures and Tables

**Table 1:** The deposition and post-deposition annealing parameters of various samples.

Sample ID	V Target Power (W)	Sn Target Power (W)	O_2_ Partial Pressure	Deposition Temperature (°C)	Post-Annealing Environment	Atomic Compositions
S0 (Torr)	50 (RF)	6	3%	Room Temperature	O_2_	V_0.40_Sn_0.1_O_0.5_
S1 (AJA International)	200 (DC)	0	5%	450	N_2_	V_0.53_O_0.47_
S2 (AJA International)	200 (DC)	10	5%	450	N_2_	V_0.46_Sn_0.03_O_0.51_
S3 (AJA International)	200 (DC)	30	5%	450	N_2_	V_0.37_Sn_0.13_O_0.5_

**Table 2:** Thickness and surface roughness of samples before and after annealing.

Sample ID	Annealing Environment	Thickness (nm)	*R_a_* (nm)	*R_S_* (nm)
S0 as deposited	None	151.2	2.63	3.5
S0 after annealing	O_2_	645	139	180
S2 as deposited	None	120	6.9	8.4
S2 after annealing	N_2_	135	7.07	17.3

**Table 3:** The resistivity of annealed samples measured at room temperature.

Sample ID	Sn%	Phase	Annealing Environment	Resistivity (Ω⋅cm)
S1	0	VO_2_	N_2_	6.19 × 10^−2^
S2	3%	VO_2_	N_2_	7.3 × 10^−2^
S3	13%	VO_2_ and SnO_2_	N_2_	2.47 × 10^−1^
S0	10%	V_2_O_5_ and SnO_2_	O_2_	5.24

**Table 4:** Comparison of properties of various materials used for microbolometer applications.

Materials	Bandgap (eV)	Resistivity (Ω⋅cm)	TCR (%/K)	Noise Voltage PSD	Reference
V_0.46_Sn_0.03_O_0.51_	0.62	0.073	−4.08	3.66 × 10^−14^ V^2^/Hz at 1 Hz to 1.77 × 10^−16^ V^2^/Hz at 100 Hz	Current work
V_2_O_5_/V	N/A	0.047	−3.18	1.32 × 10^−9^ V^2^/Hz at 12 Hz	Abdel-Rahman et al. [8,46]
VO_x_	N/A	10^−3^ to 6.8 × 10^4^	0 to −4	N/A	Gauntt et al. [34]
VO_x_	N/A	10^−2^	−2.54	N/A	Scott et al. [42]
V_x_Mo_y_O_z_		~1 Ω·cm	−1.7 to −2.2		Ozcelik et al. [36]
V_x_Ti_y_O_z_		~1 Ω·cm	−1.6		
V_x_Zr_y_O_z_		~1 Ω·cm	−1.9 to −2.7		
a-Si	N/A	5000	−3.7	N/A	Voshell et al. [5]
SiGeO	0.75 to 0.89	N/A	−3.5 to −8.7	N/A	Rana et al. [6]
GeSiSnO	1.03	1.464 × 10^−3^	−3.26	1.76 × 10^−11^ V^2^/Hz at 2 Hz to 2.28 × 10^−12^ V^2^/Hz at 10 Hz	Vadakepurathu et al. [13]
TiO_2_	N/A	0.01 to 10	−2.8	N/A	Kwon et al. [9]

## Data Availability

Data presented in this study are available on request from the corresponding author due to privacy.
